# Concomitant Lumbar Stenosis and Aortic Pseudoaneurysm: A Case Report

**DOI:** 10.7759/cureus.822

**Published:** 2016-10-09

**Authors:** Christoph Fuchs, Thomas E Niemeier, William E Neway, Sakthivel Rajan Rajaram Manoharan

**Affiliations:** 1 Orthopaedic Surgery, UAB School of Medicine; 2 Orthopaedics, Hughston Clinic

**Keywords:** tlif, minimally invasive spine surgery, abdominal aortic pseudoaneurysm, vertebral erosion, lumbar stenosis

## Abstract

Aortic pseudoaneurysm can create a constellation of symptoms that can mimic lumbar back pain. There are rare but well-documented reports of aortic pathology (aneurysms, pseudoaneurysms, and chronic contained aneurysm ruptures) eroding into the vertebral column causing neural compression. We report a case of a rapidly progressive aortic pseudoaneurysm in a patient with pre-existing lumbar spine pathology which had the potential for catastrophic intraoperative bleeding during a minimally invasive surgery (MIS) using the transforaminal lumbar interbody fusion (TLIF) technique. Postoperatively, the patient’s radicular pain resolved but her back pain remained. Further workup identified the pseudoaneurysm and the patient subsequently underwent open vascular repair. In this report, we highlight a lesser known mimicker of lumbar back pain.

## Introduction

Symptomatology related to lumbar stenosis and degeneration is well known in the medical community. For those treating this condition, it is important to recognize other conditions that can mimic low back pain—especially those that are life threatening. Herein, we present the case of a diagnostically challenging patient with known severe lumbar stenosis and a concomitant infrarenal aortic pseudoaneurysm. Written informed consent was obtained as per institutional protocol for the review and publication of this case report.

## Case presentation

**Figure 1:**
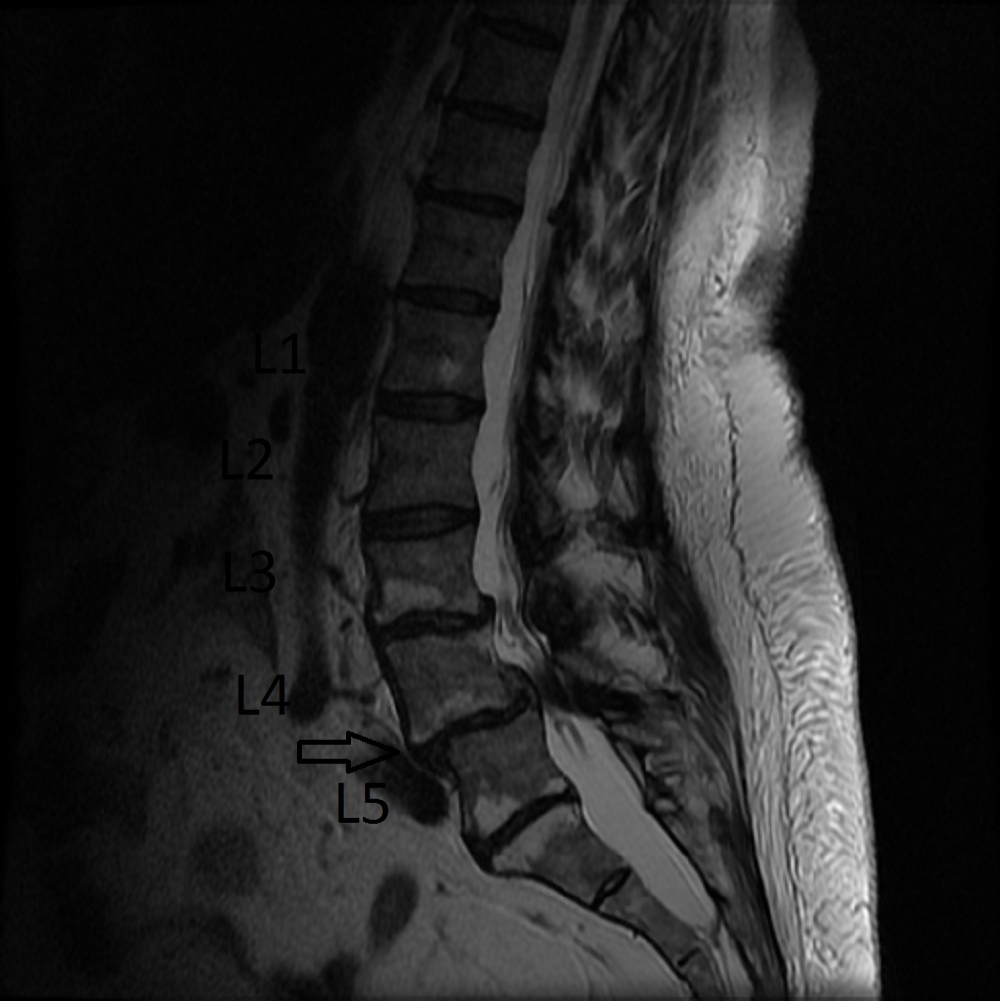
Pre-op Midsagittal Section: MRI of Lumbar Spine

**Figure 2:**
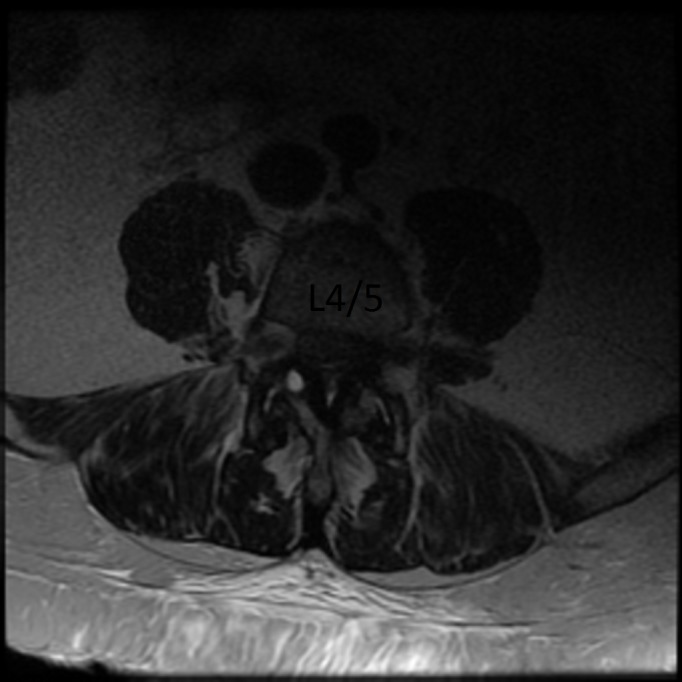
Pre-op Axial Section L4-5: MRI of Lumbar Spine

A 59-year-old female presented to a spine clinic with lumbar back pain, neurogenic claudication, and lumbar radiculopathy. Imaging was consistent with L4-5 degenerative spondylolisthesis with severe lumbar stenosis (Figures 1-2).

Due to her complex medical comorbidities including obesity, chronic obstructive pulmonary disease (COPD) on home oxygen, hypothyroidism, and myasthenia gravis on prednisone, she was initially treated nonoperatively. Approximately one month later, she was transferred to our institution after being admitted at an outside hospital for intractable low back pain and inability to ambulate. The patient was medically optimized and subsequently underwent a MIS TLIF surgery for L4-5.

**Figure 3:**
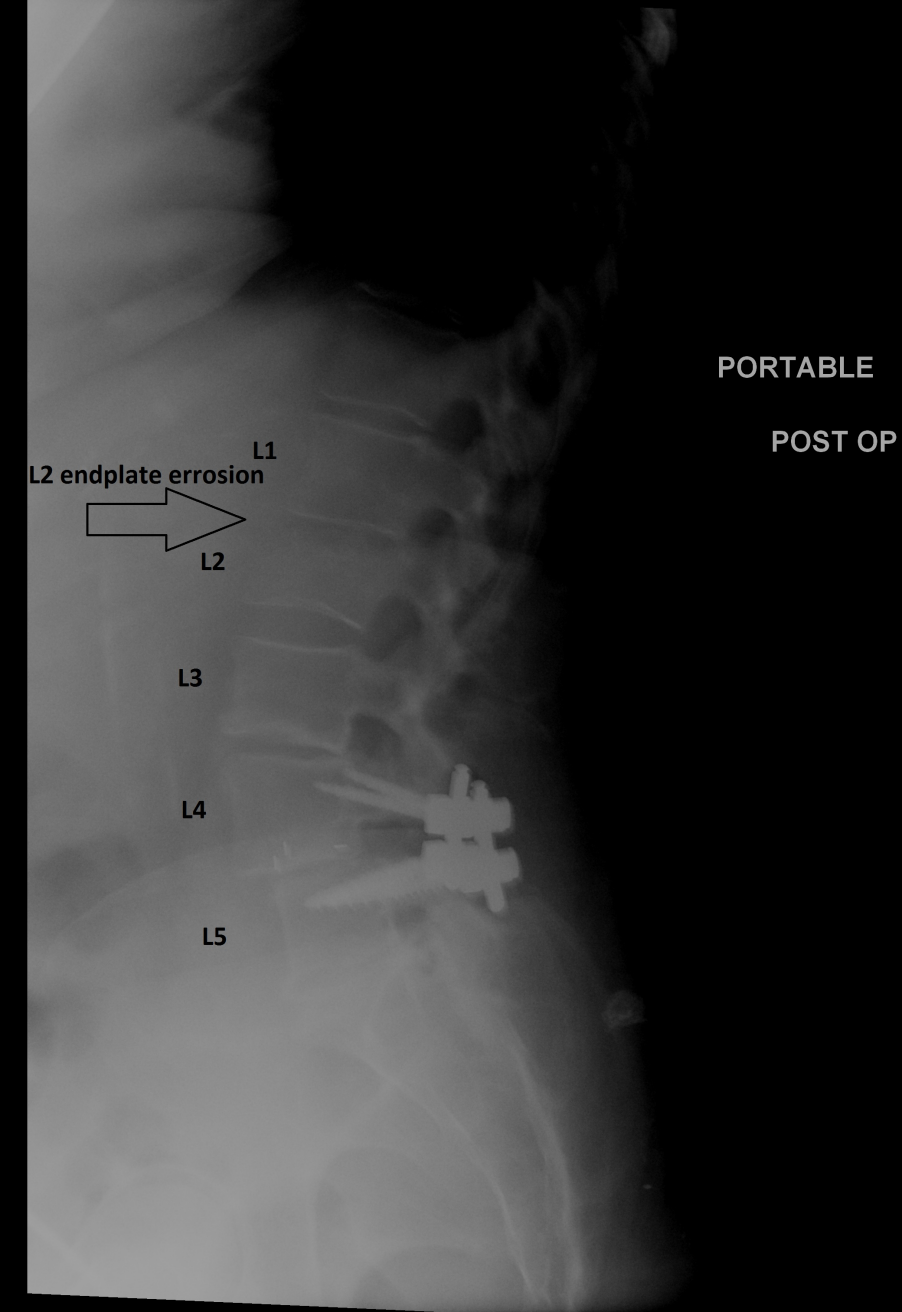
Postoperative X-rays with L2 Erosive Changes

Postoperatively, the patient’s radicular lower extremity pain resolved but the pain in her low back did not. Routine postoperative radiographs demonstrated new erosion of the anterior-inferior L2 vertebral body (Figure 3) suggestive of an acute infectious process.

**Figure 4:**
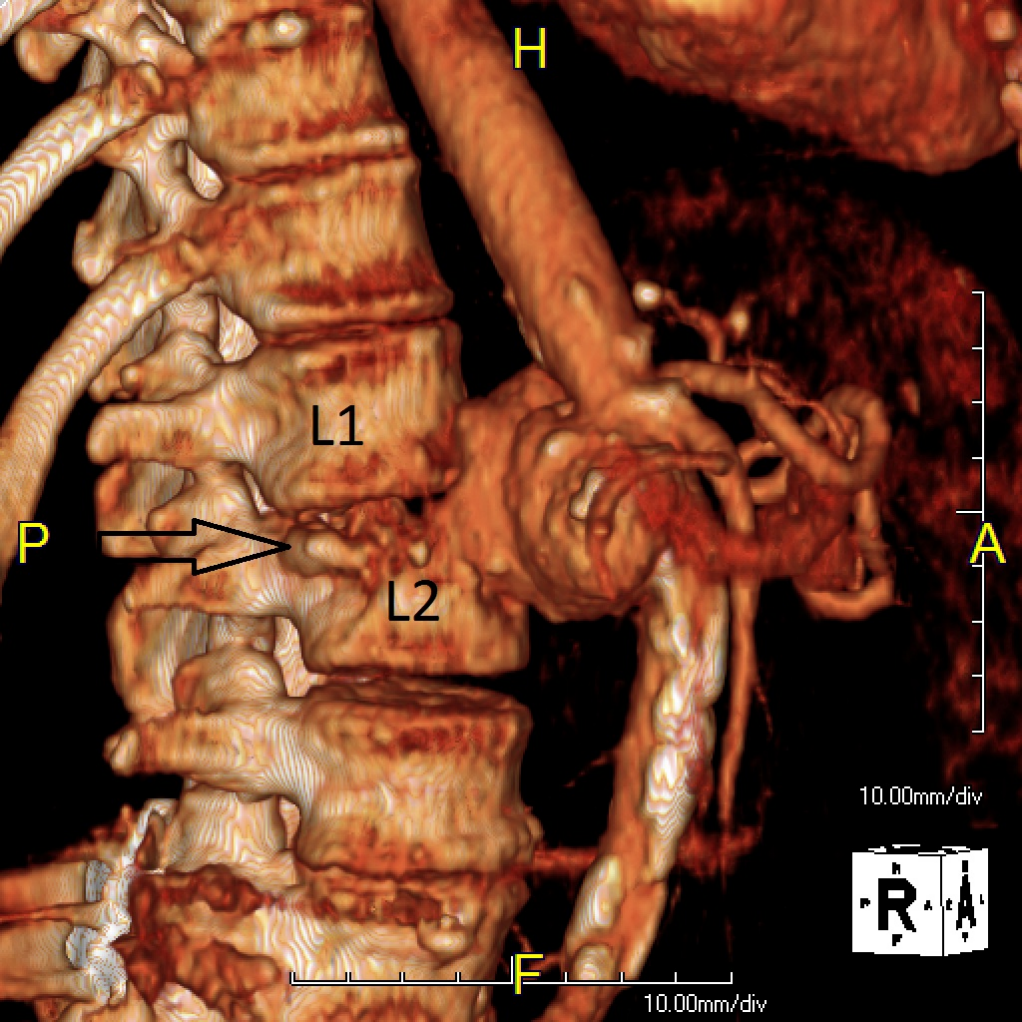
CT Angiogram 3D Reconstruction

Further advanced imaging revealed a 5.2 cm infrarenal aortic pseudoaneurysm extending into the L2 vertebral body (Figure 4).

Vascular surgery was consulted and predicted a 100% risk of mortality if the aneurysm was not addressed urgently. The patient subsequently underwent open aortic replacement with a Dacron graft. Intraoperative cultures and biopsy failed to demonstrate an infectious etiology. After a prolonged hospital stay, the patient expired secondary to multisystem organ failure.

## Discussion

Lumbar decompression and fusion surgeries have recently become one of the most common procedures performed in the United States. Advances in surgical techniques and equipment have allowed improved outcomes while decreasing morbidity of the procedure. Recently, TLIF has gained in popularity as it can be inserted with minimally invasive techniques and provides restoration disc space while predictably relieving neural compression [1].

Indications for lumbar fusion surgery are fairly well established [2]. As a large percentage of the population will develop lumbar back pain during their lifetime, patient selection prior to surgical intervention is of the utmost importance. Thus, it is important for the spine surgeon to be knowledgeable of conditions that mimic lumbar back pain and radiculopathy. One such condition that is infrequently encountered is central vascular disease. Pathology of the aorta can result in a wide variety of clinical conditions secondary to erosions of the vertebral bodies and neural compression. Multiple case reports have described axial back pain and radiculopathy as the presenting symptoms [3-5]. Not only do these cases present a diagnostic challenge, but given their ability to erode into the spinal column and occasionally the spinal cord [6], they have the potential for catastrophic intraoperative bleeding if unknowingly encountered by a spine surgeon.

Aortic pathology is divided into several subtypes. True aortic aneurysms, defined as involving all three layers of the aortic wall, erode into vertebrae in less than seven percent of cases and occasionally cause back pain and spinal instability [7]. False or pseudoaneurysms are outpouchings of the vessel wall that do not involve all three vessel layers but can similarly erode into the spinal column [3, 8]. Contained aneurysm ruptures, first described by Szilagyi in 1961 [9], occur when the aneurysm rupture takes place in a location where the surrounding structures, such as the vertebral column and paraspinal muscles, temporarily tamponade the bleeding. Mycotic aneurysms occur either through direct vessel wall infection or in association with contiguous discitis [10]. Regardless of classification, the natural progression of all subtypes is enlargement with the potential for rupture.

Our patient had a well-defined indication for surgery with severe spinal stenosis secondary to L4-5 degenerative spondylolisthesis. Her numerous medical comorbidities initially made her less than an ideal surgical candidate but her worsening back pain limiting ambulation prompted surgical intervention. It is possible that her acute worsening pain was due to confined rupture of her pseudoaneurysm and not her lumbar spine. On retrospective review, there was no evidence of aortic aneurysm on a magnetic resonance imaging (MRI) scan obtained two months prior, or vertebral erosions on X-rays four weeks prior to admission to the hospital, indicating rapid expansion of the aneurysm. Fortunately, the location of vertebral and disc space erosion was several levels superior to her lumbar stenosis. Otherwise, catastrophic intraoperative bleeding would likely have occurred. To our knowledge, there are no reports of an unfortunate surgeon encountering previously unknown aortic pathology that has eroded into the operative field.

## Conclusions

It is important for a spine surgeon to recognize vascular disease as a rare but possible etiology of both axial and radicular pain. When obtaining advanced imaging of the spine, windows need to be wide enough to allow visualization of the aorta and paraspinal musculature. Careful scrutiny of suspicious vertebral body endplates and an enlarged, calcified aorta on radiographs should prompt further diagnostic workup. Computed tomography (CT) angiogram is the imaging modality of choice for aortic disease. Discovery of all subtypes of aortic pathology should trigger a prompt referral to a vascular surgeon.
